# Biomarker responses and accumulation of polycyclic aromatic hydrocarbons in *Mytilus trossulus* and *Gammarus oceanicus* during exposure to crude oil

**DOI:** 10.1007/s11356-020-07946-7

**Published:** 2020-02-20

**Authors:** Raisa Turja, Steinar Sanni, Milda Stankevičiūtė, Laura Butrimavičienė, Marie-Hélène Devier, Hélène Budzinski, Kari K. Lehtonen

**Affiliations:** 1 Marine Research Centre, Finnish Environment Institute, Agnes Sjöbergin katu 2, FI-00790 Helsinki, Finland; 2 NORCE - Norwegian Research Centre, Mekjarvik 12, N-4072 Randaberg, Norway; 3 Faculty of Science and Technology, Department of Mathematics and Natural Science, University of Stavanger, N-4036 Stavanger, Norway; 4 Nature Research Centre, Institute of Ecology, Akademijos str. 2, LT-08412 Vilnius, Lithuania; 5 Laboratory of Physico- and Toxico-Chemistry of the Environment (LPTC), University Bordeaux 1, Oceanic and Continental Environments and Paleoenvironments (EPOC, UMR 5805 CNRS), 351 cours de la Libération, F-33405 Talence, France

**Keywords:** Bioaccumulation, Biomarkers, Crude oil, Gammarids, Mussels, Polycyclic aromatic hydrocarbons

## Abstract

In the brackish water Baltic Sea, oil pollution is an ever-present and significant environmental threat mainly due to the continuously increasing volume of oil transport in the area. In this study, effects of exposure to crude oil on two common Baltic Sea species, the mussel *Mytilus trossulus* and the amphipod *Gammarus oceanicus*, were investigated. The species were exposed for various time periods (*M. trossulus* 4, 7, and 14 days, *G. oceanicus* 4 and 11 days) to three oil concentrations (0.003, 0.04, and 0.30 mg L^−1^ based on water measurements, nominally aimed at 0.015, 0.120, and 0.750 mg L^−1^) obtained by mechanical dispersion (oil droplets). Biological effects of oil exposure were examined using a battery of biomarkers consisting of enzymes of the antioxidant defense system (ADS), lipid peroxidation, phase II detoxification (glutathione *S*-transferase), neurotoxicity (acetylcholinesterase inhibition), and geno- and cytotoxicity (micronuclei and other nuclear deformities). In mussels, the results on biomarker responses were examined in connection with data on the tissue accumulation of polycyclic aromatic hydrocarbons (PAH). In *M. trossulus*, during the first 4 days of exposure the accumulation of all PAHs in the two highest exposure concentrations was high and was thereafter reduced significantly. Significant increase in ADS responses was observed in *M. trossulus* at 4 and 7 days of exposure. At day 14, significantly elevated levels of geno- and cytotoxicity were detected in mussels. In *G. oceanicus*, the ADS responses followed a similar pattern to those recorded in *M. trossulus* at day 4; however, in *G. oceanicus*, the elevated ADS response was still maintained at day 11. Conclusively, the results obtained show marked biomarker responses in both study species under conceivable, environmentally realistic oil-in-seawater concentrations during an oil spill, and in mussels, they are related to the observed tissue accumulation of oil-derived compounds.

## Introduction

Oil pollution continues to be a significant marine environmental threat. In the Baltic Sea, the occurrence of a major oil spill is considered to cause widespread damage to the local ecosystem. The specific characteristics of the Baltic Sea including semi-closedness with slow water exchange, a fragmented and shallow coastline, harsh winter conditions, and low biodiversity make it highly vulnerable to oil pollution (Rousi and Kankaanpää 2012). For example, in the Gulf of Finland, the annual transportation of crude oil and refined products is currently 160–170 million tonnes and the overall oil tanker traffic is constantly growing, thus increasing the risk of a major accident in the area (Hänninen and Rytkönen 2004; Lehikoinen et al. 2015). Preparedness for efficient response actions in case of a large oil spill includes good understanding of the potential biological effects of oil on local biota (Martínez-Gómez et al., 2010), which is also essential in the designing of effective pre- and post-spill monitoring strategies (Marigómez et al., 2013).

Crude oils transported along global shipping routes have a highly variable chemical composition and consist of thousands of different compounds influencing its environmental fate and toxicity (NRC 2003). Polycyclic aromatic hydrocarbons (PAHs) represent a highly toxic group of compounds that are readily taken up by marine organisms directly from the water through body surfaces or gills, or through the diet, inducing acute and long-term toxic effects (Hylland 2006; Martinez-Gomez et al. 2010). In addition to parent PAH compounds, crude oils contain other hydrocarbons such as thiophenes (sulfur-containing analogues of PAHs) and alkylaromatic compounds such as methylphenanthrenes and methylnaphthalenes (Neff et al. 2011; Pampanin and Sydnes 2013). These compounds have shown geno- and cytotoxic potential in human liver cells (Amat et al. 2004), and, although not widely studied, have been shown to bioaccumulate efficiently and to elicit sublethal effects also in marine organisms (Kropp and Fedorak 1998; Booth et al. 2007; Rowland et al. 2001).

During the last decades, the approach of determining actual biological effects of contaminants on biota by using various types of biomarkers has been widely elaborated and applied both in laboratory and field studies (van Der Oost 2003; Garmendia et al. 2011); Vethaak et al. 2017; Sanni et al. 2017; Beyer et al. 2017). Post-accident monitoring campaigns in relation to marine oil spill disasters such as “Exxon Valdez” (Alaska, USA) in 1989 and “Prestige” (Galicia, Spain) 2002 still showed detectable biological effects in organisms after several years from the accidents when tissue accumulation of hydrocarbons could not be observed anymore (Esler et al. 2010). In the Baltic Sea, biomarker studies on some key species have shown that many of them are sensitive to various pollutants commonly found in the marine environment, including compounds resulting from oil contamination (Baršienė et al. 2006a; Baršienė et al. 2012; Turja et al. 2013, 2014b; Lehtonen et al. 2016).

Apart from the well-known AhR receptor-mediated toxicity of PAHs, major threats to cellular integrity are generated by the increased production of reactive oxygen species (ROS) during the biotransformation processes, leading to an imbalanced redox state where ROS are not sufficiently neutralized by the antioxidant defense system (ADS) and cause damage to macromolecules such as lipids, proteins, and DNA (Baussant et al. 2009; Regoli and Giuliani 2014). Moreover, biotransformation of PAHs is known to produce highly reactive byproducts such as DNA-binding metabolites, resulting in geno- and cytotoxic effects (Le Dû-Lacoste et al. 2013). PAHs are known to induce the ADS and to cause oxidative damage in aquatic organisms through the production of ROS (Regoli and Giuliani 2014). Organisms use catalase (CAT), superoxide dismutase (SOD), glutathione reductase (GR), and glutathione peroxidase (GPx) as the main enzymatic antioxidants that provide cellular defense against endogenous and exogenous ROS. Membrane damage caused by ROS can be detected by measuring lipid peroxidation (LPO). Regarding detoxification of organic contaminants, the activity of glutathione *S*-transferase (GST), involved in phase II (conjugation), is a widely used biomarker in organisms exposed to PAHs (e.g., Kopecka et al. 2006; Richardson et al. 2008; Turja et al. 2014b). Exposure to oil compounds has shown to cause neurotoxic effects, e.g., inhibition of acetylcholinesterase (AChE) enzyme activity (Maisano et al. 2017). Moreover, severe geno- and cytotoxic effects have been reported in response to oil contamination, including the formation of micronuclei (MN) and other nuclear deformities (Baršienė et al. 2012).

Selection of suitable organisms is an important step in the designing of biomonitoring programs. Mussels (especially of the genus *Mytilus*) and other bivalve molluscs are used extensively in biomonitoring to detect both the bioaccumulation of hydrocarbons and biological responses in relation to oil contamination (e.g., Cajaraville et al. 2000; Hylland et al. 2008; Turja et al. 2013). Their sessile, filter feeding lifestyle and generally low enzymatic degradation rates of organic contaminants render them capable of accumulating high levels of organic molecules, including PAHs. Crustacean amphipods, and especially species belonging to the genus *Gammarus*, are also considered as excellent bioindicator species since they are widespread over large salinity and habitat ranges (Whiteley et al. 2011) and respond to various types of environmental contamination including PAH pollution (e.g., Turja et al. 2014b).

Information on biological effects of crude oil in Baltic Sea species is scarce. The objective of the present study was to investigate the effects of exposure to crude oil on two Baltic Sea species, the mussel *Mytilus trossulus* and the amphipod *Gammarus oceanicus*, by applying the biomarker methods mentioned above. The species were exposed for various time periods to a range of oil concentrations obtained by mechanical dispersion. The results on biomarker responses were examined in connection with the PAH tissue accumulation data obtained during the experiments.

## Materials and methods

The experimental animals were collected in September 2009 from two sites on the SW coast of Finland (Baltic Sea) where at 5 m depth the sea water salinity was 6.0 and temperature was 14 °C. *M. trossulus* were collected by SCUBA diving in the coastal area of Hanko and *G. oceanicus* with hand nets from the outer archipelago (Granbusken skerry) near the Tvärminne Zoological Station of the University of Helsinki, considered to be a relatively clean area. The mussels were gently cleaned to remove any sessile organisms growing on their shells. After collection, the organisms were transported within 48 h in aerated, thermo-insulated boxes filled with water from the collection site to the special NORCE facilities (formerly International Research Institute of Stavanger; IRIS), Norway, for the oil exposure experiments. The desired brackish water salinity of 6.0 (characteristic for the Baltic Sea in SW Finland) used in the experiment was obtained by mixing natural seawater of (salinity 32) with tap water (not containing chlorine). The seawater used during these experiments was directly pumped from the fjord at 78 m depth and was sand filtered before use in the laboratory. The animals were acclimatized for 7 days prior to the experiment in flow-through 350 L tanks. During this period, the mussels were fed every other day with algal cells (Instant Algae®) and the amphipods with tiny pieces of shrimp meat. Sampling (day 0) for mussels and gammarids was carried out after the acclimatization period.

### Experimental design

A continuous flow system was used to create a dispersion of crude oil (type Arctic Oil) in seawater. The oil was injected into seawater under pressure and passing through a valve to form small oil droplets (Sanni et al. 1998). Nominal concentration of injected oil, 5 mg L^−1^ (7 L min^−1^ seawater and 0.042 μL min^−1^ oil), was used to make the dilutions used in the exposure concentrations. Brackish seawater was constantly prepared in an open tank in order to remove chlorine. The 5 mg L^−1^ oil dispersion was pumped into four mixing glass flasks where it was diluted to the nominal concentrations of 0.015, 0.120, and 0.750 mg L^−1^ with brackish water (Table 1), hereafter named as experimental groups of low (Lo), medium (Med), and high (Hi) oil concentration. The dispersed oil was constantly pumped into the experimental system with adjusted flow rates (Watson Marlow 520S pump, Teflon tubes [Swagelok]) for each treatment. Water flow rate from the mixing bottles into the aquaria was adjusted to 700–800 mL min^−1^. The experiment was carried out in a temperature-adjusted hall with a constant temperature of 14 °C and a 12:12-h daylight rhythm.

**Table 1:** Measured PAH compounds in batch of Arctic crude oil used in the exposures

Compound	μg g^−1^ oil (ppm)
Naphthalene	985
C1-naphthalene	2694
C2-naphthalene	4196
C3-naphthalene	2834
Sum 2-ring PAHs	10,709
Acenaphthylene	mi *<
Acenaphthene	mi *<
Fluorene	73
Phenanthrene	170
Anthracene	*<
C1-Phen/Anthr	275
C2-Phen/Anthr	307
Sum 3-ring PAHs	825
Dibenzothiophene	25
C1-dibenzothiophene	69
C2-dibenzothiophene	70
Sum DBTs	164
Fluoranthene	6
Pyrene	5
Benzo(a)anthracene	3
Chrysene/triphenylene	9
C1-chrysene	21
C2-chrysene	24
Sum 4-ring PAHs	68
Benzo(b,j)fluoranthene	4
Benzo(k)fluoranthene	*<
Benzo(a)pyrene	2
Sum 5-ring PAHs	6
Indeno(1,2,3-cd)pyrene	*<
Benzo(g,h,i)perylene	*<
Dibenzo(a,h)anthracene	*<
Sum 6-ring PAHs	0
Total measured PAHs	11,772
Percentage of oil wt.	1.2%

mi *<: matrix interference, *<: below quantification limit

*M. trossulus* were exposed in four aquaria of 100 L, one for each treatment concentration, for 4, 7, and 14 days. At the beginning of the experiment, a total of 250 adult mussels sized 2.5–3.0 cm were added to each aquarium. At each sampling time 50–70 mussels were randomly removed from each aquarium and, again, randomly divided for the different analyses. Adult *G. oceanicus* were exposed in seven replicate aquaria (30 L) per treatment concentration for four and 11 days. At the beginning of the experiment, 80 *G. oceanicus* individuals of 1–1.5 cm in length were placed in each aquarium. Five pooled samples of five individuals were removed from each aquarium for analyses after 4 days and three pooled samples after 11 days of exposure. The bottom of the aquaria was cleaned every day and any dead individuals found were removed. The flow-through system was stopped once a week to clean the mixing bottles and oil tubes with hot tap water and rinsed with brackish water to avoid oil blocking the system. Animals were not fed during the experiment to avoid algal culture-related changes in clean water (controls) compared to the water with oil (oil exposure treatments) and thus result in difficult feeding-related differences between the treatments. Moreover, non-feeding was not expected to affect the results markedly due to the reason that Baltic Sea organisms have their energy reserves filled up at this time of the year being therefore naturally adapted to fasting occurring during the long winter period.

### Chemical analysis of hydrocarbons in water and in mussel tissues

Water concentrations of 26 different PAH compounds were analyzed in the laboratory, based on a standard protocol (EPA 610) with previously described modifications (Jonsson et al. 2004). Water samples were collected weekly from the 5 mg L^−1^ solution (see more details from a parallel experiment by Ingvarsdóttir et al. 2012) and once from the exposure aquaria of *M. trossulus*. Limit of quantification (LOQ) was set to approximately 0.005 μg L^−1^ for each PAH component.

Analyses of PAH and other selected compounds in the tissues of mussels were carried out at the LPTC Université Bordeaux I using established protocols of the laboratory. Pooled samples consisting of whole soft tissue of 30 mussels were used for the analyses. The samples were freeze-dried (Edwards Super Modulyo freeze dryer) and pulverized with a grinding mortar. Extraction and quantification protocols for PAHs have been described elsewhere (Baumard et al. 1997). All steps of the analytical protocol were validated in terms of reproducibility and accuracy; procedural blanks were systematically checked and certified reference mussel tissue (1974a NIST) were analyzed together with the actual samples (Tapie et al. 2008; Thompson and Budzinski 2000). The obtained recoveries ranged between 70 and 20% with coefficient of variation < 20%. The detection limits of individual compounds in mussel tissues were in the range 0.1–1.0 ng g^−1^ dry weight.

### Biomarker measurements

Most of the biomarkers were analyzed only in *M. trossulus*. In *G. oceanicus*, the analyzed parameters included the ADS biomarkers and biotransformation phase II activity (GST).

#### ADS, biotransformation phase II activity, and neurotoxicity

Enzymatic activities of CAT, GR, GPx, and SOD as well as the level of LPO were measured for the ADS response. GST was measured for biotransformation phase II activity, and AChE was measured to assess neurotoxicity. For the enzymatic assays, digestive glands of mussels (*n* = 15) were homogenized in potassium phosphate buffer (100 mM, pH 7.4) and gills (*n* = 15) in sodium phosphate buffer (200 mM, pH 7.0) containing 0.1% Triton-X. Pooled samples of whole *G. oceanicus* individuals were homogenized in 50 mM potassium phosphate buffer including 2 mM EDTA (pH 7.5).

GST activity was estimated by measuring the formation rate of the conjugated substrate (chlorodinitrobenzene [CNDB]-glutathione [GSH]) at 340 nm (Habig et al. 1974). Final concentrations of 1 mM CNDB (Sigma 237329) and 1 mM GSH (Sigma G6529) in potassium phosphate buffer (100 mM, pH 7.0) were used in the reaction. CAT activity was measured as CAT mediated degradation of hydrogen peroxide (H_2_O_2_) at 240 nm (Claiborne 1985). The reaction mixture contained 4.3 μM H_2_O_2_ (Fluka 95302) in potassium phosphate buffer (100 mM, pH 7.0). GR activity was measured indirectly as consumption of NADPH in the reduction of oxidized glutathione (GSSG) (Carlberg and Mannervik 1975). The reaction mixture contained 2 mM EDTA (Sigma E5134), 0.5 mM GSSG (Sigma G4376), and 0.1 mM NADPH (Sigma N7505) in potassium phosphate buffer (100 mM, pH 7.5). GPx activity was measured by the consumption of NADPH in a glutathione reductase-coupled enzyme assay according to the method described by Flohe and Gunzler (1984). The reaction mixture contained 20 mM GSH (Sigma G6529), 2 mM NADPH (Sigma N7505), 10 U mL^−1^ GR (Sigma G3664), 6 mM H_2_O_2_ (Fluka 95302) in potassium phosphate buffer (100 mM, pH 7.5) with 2 mM EDTA (Sigma E5134), 1 mM DTT (Sigma D9779), and 1 mM NaN_3_ (Sigma S2002). SOD activity was defined as the amount of enzyme required to inhibit the rate of reduction of cytochrome c by 50% and measured at 550 nm (Mccord and Fridovic, 1969). The reaction mixture contained 1 mM EDTA (Sigma E5134), 0.1 M NaOH, 4.7 mM xanthine (Sigma X7375), 0.2 M cytochrome *c* (Sigma), and 6.7 mU xanthine oxidase (Sigma X1875) in sodium phosphate buffer (50 mM, pH 7.8). SOD (Sigma S7531) dilutions from 0 to 1.5 U mL^−1^ were made to create the standard curve. Levels of LPO were measured as the generation of thiobarbituric acid reactive substances (TBARS) (Ohkawa et al. 1979). The reaction mixture contained 0.24 M trichloroacetic acid (Riedel de Haën 33731), 60mM Tris-HCl with 0.1 mM DTPA, and 16 mM 2-thiobarbituric acid (Sigma T5500). The amount of TBARS was measured by reading absorbance at 535 nm. Analyses of AChE activity were performed from gill samples as described in Bocquené and Galgani (1998) with modifications as in Leiniö and Lehtonen (2005). AChE activity values are expressed as equivalents of acetylthiocholine (ACTC) hydrolyzed (nmol ACTC min^−1^ mg protein^−1^), with 1 ΔO.D. corresponding to the hydrolysis of 75 nmol of ACTC.

All the enzymatic assays, LPO, and protein content (Bradford 1976) used for the calculation of specific enzymatic activities were measured in 96-well microplates using the TECAN Infinite 200 (TECAN) spectrophotometer with the Magellan software.

#### Morphometric condition of *M. trossulus*

Condition index was (CI) determined in mussels (*n* = 15) using the formula CI = (soft tissue dry weight [mg] / shell length [mm]^2^) × 100. The weight change in total dry soft tissue of mussels in the different treatments and time points was determined for a standard size individual of 30 mm shell length using the allometric regression *W* = *aL*^*b*^, where *W* = dry weight, *L* = length, and *a* and *b* intercept and slope, respectively.

#### Geno- and cytotoxicity biomarkers in *M. trossulus*

Mussel gills were analyzed after 14 days of exposure for the selected geno- and cytotoxicity parameters including micronuclei (MN), nuclear buds (NB), binucleated cells (BN), and fragmented apoptotic cells (FA). MN are extra-nuclear bodies that contain damaged chromosome fragments and/or whole chromosomes that are not incorporated into the nucleus after cell division. Frequency of BN is an indicator of abnormal cell division due to the disturbed cytokinesis while FA indicates changes in the apoptotic rate since elimination of cytogenetic damage by the apoptosis is a key process occurring at different rates in organisms (Fernandez et al. 2011; Mičić et al. 2002). Preparation of slides and investigation of gill cells was carried out following the method described earlier (Baršienė et al. 2004, 2006b). The stained slides were analyzed under bright-field Olympus BX51 microscopes (Tokyo, Japan) using an immersion objective (1000×). Two thousand cells with intact cellular and nuclear membranes per mussel were evaluated using blind scoring. The frequency of the different nuclear abnormalities was expressed as the number of occurrences per 1000 cells scored. The formation of MN and NB were assessed as genotoxicity endpoints, and FA, BN cells as cytotoxicity endpoints.

#### Data integration and statistical analysis

The integrated biomarker index (IBR; Beliaeff and Burgeot 2002) is a simple mathematical tool based on the standardization of the different biomarker values and finally summing up each two neighboring biomarkers. To compare all biomarkers with each other, IBR was calculated for three different biomarker arrangements. The final IBR value is the mean value of these three arrangements. Here, a modification of the method where the index values are given divided by the number of biomarkers included in the calculations (IBR/n; Broeg and Lehtonen 2006) was applied. In *M. trossulus*, seven biomarkers (CAT, GST, GR, SOD, and LPO measured in the digestive gland, AChE in gills, and CI) were used to calculate the IBR for 4 and 14 days of exposure. Since LPO and CI were not measured at day 7, they were left out leaving five biomarkers for IBR calculation at that time point. GST and CAT measured in gills were left out, because including them twice would emphasize these responses too much in the index value. Geno- and cytotoxicity were measured only at day 14 and for that reason they were left out from the IBR making it clearer to study those responses separately from index value calculated mainly with enzymatic responses.

For *G. oceanicus*, all the five measured biomarkers CAT, GST, GPx, SOD, and LPO were included in the calculation of the IBR for 4 and 11 days of exposure.

All data were tested for the normality with the Kolmogorov–Smirnov test and homogeneity of variance with Bartlett’s test. One-way ANOVA (*F* statistics) followed by Bonferroni corrected pairwise *t* test were used for normally distributed data and Kruskal–Wallis for non-parametric data (*H* statistics). Program R was used to calculate the statistics.

## Results

### General observations

In mussels, byssal thread formation was markedly decreased in higher oil concentrations. This was quantified by visual inspection and gently touching the mussels during the cleaning of the aquaria. In the control and Lo treatments, 90–95% of the mussels were properly attached to the bottom with byssi, while at Med their share was 50% and at Hi only 5% (data not shown). Mussels kept their valves open in all treatments and were apparently filtering the water actively. However, by visual inspection, the amount of produced feces was markedly smaller in the Hi treatment. Average mortality in the control aquarium was three individuals per day and in the oil-exposed aquariums five individuals per day. In amphipods, average mortality in each control aquarium was one individual per day and in the oil treatments two individuals per day per aquarium.

### PAHs in the exposure water

Analysis of the oil used in the exposure showed that it contained 1.2% of PAHs analyzed as 26 PAH constituents (Table 1). This is comparable to approximate per cent content of 1.5% PAH in crude oil as calculated from Neff [2002]. Analysis of water concentrations of the 26 PAHs resulted in the detection of eight compounds (Table 2). PAH concentrations measured in the exposure treatments showed reasonably good agreement with the target dilution range of the prepared crude oil exposure stock solution, being 0.040, 0.498, and 3.555 μg L^−1^ in the Lo, Med, and Hi treatments, respectively (Table 2). The actual PAH concentrations based on dilution alone could thus have been expected to be 2–4 times higher than that measured in the treatments; however, in oil exposure experiments lower concentrations in the water phase are common due to the hydrophobic oil substances being attached to tubes, tank walls and even the test organisms (shells of mussel). On the other hand, PAHs loading onto these surfaces could be a secondary supplier, thus altering their levels into the seawater during the experiment (e.g., Giannapas et al. 2012). However, these issues are difficult to assess with precision. Under the assumption that 1.2% of the crude oil used were PAHs, an estimate of actual oil exposure concentration would correspondingly be approximately 0.003, 0.04, and 0.30 mg L^−1^.

**Table 2:** Water concentrations (μg L^−1^) of selected PAHs detected in the prepared crude oil exposure stock solution (5 mg L^−1^, three replicate measurements; from Ingvarsdóttir et al. 2012) and in the exposure aquaria after dilution to the desired exposure concentrations of Lo, Med, and Hi (one measurement, present study) in the flow-through exposure system

	Crude oil stock solution	Exposure concentrations of crude oil		
	5 mg L^−1^	Lo	Med	Hi
Naphthalene	3.9 ± 1.4	0.003	0.031	0.207
C1-naphthalene	11.6 ± 4.2	0.008	0.116	0.771
C2-naphthalene	17.7 ± 5.8	0.014	0.176	1.337
C3-naphthalene	13.1 ± 4.2	0.012	0.135	1.019
Fluorene	0.2 ± 0.1	nd	0.003	0.017
Phenanthrene	0.6 ± 0.2	0.002	0.009	0.045
C1-phenanthrene/anthracene	1.0 ± 0.3	0.001	0.012	0.074
C2-phenanthrene/anthracene	1.2 ± 0.4	nd	0.015	0.085
ΣPAHs	49.4 ± 16.6	0.040	0.498	3.555

*nd* not detected

C1–C3 naphthalenes occurred in up to two orders of magnitude higher concentrations compared to the other measured PAHs (fluorene, phenanthrene [PHE], and the C1- and C2-PHE/anthracene sum parameter).

### PAHs in mussel tissues

During the experiment, the exposed mussels showed tissue accumulation of most of the analyzed PAH compounds (Table 3). At the higher concentrations of Med and Hi, a clear time-dependent accumulation hydrocarbons was observed. However, similar tissue concentrations were observed in the control and Lo-treated mussels after 4 and 14 days of exposure. PAH concentration in the Lo treatment was very low, 0.04 μg L^−1^, which did not cause accumulation exceeding the observed background PAH level in tissues.

**Table 3:** *Mytilus trossulus*. PAH concentrations (ng^−1^ g^−1^ dry weight) measured in soft tissues after exposure to mechanically dispersed oil at different concentrations Lo, Med, and Hi and time periods (4 and 14 days)

	CT	Lo	Med	Hi	CT	Lo	Med	Hi
	4 days				14 days			
PAH_18_								
Naphthalene	4.80	0.70	6.30	12.10	5.10	5.70	1.90	7.30
Acenaphthylene	0.50	0.30	0.20	0.50	0.20	0.20	nd	0.10
Acenaphtene	0.60	0.20	0.40	0.70	0.30	0.30	nd	0.80
Fluorene	1.50	1.30	2.10	7.60	1.60	1.60	3.40	7.80
Phenanthrene (PHE)	69.30	69.50	85.50	145.10	82.60	75.40	98.90	177.10
Anthracene	4.00	3.20	3.30	3.20	2.60	2.00	2.40	4.30
Fluoranthene (FLU)	31.20	29.80	38.50	64.40	27.00	26.70	50.90	89.10
Pyrene (PYR)	72.20	62.80	67.60	92.30	55.20	44.80	71.90	101.20
Benzo[a]anthracene	5.60	5.50	8.60	17.80	5.20	3.70	11.30	26.40
Triphenylene+chrysene	10.70	11.80	32.10	95.60	8.80	10.20	58.90	181.80
Benzo[b,j,k]fluoranthene	2.70	4.70	9.50	20.10	6.90	7.00	21.20	39.80
Benzo[e]pyrene	2.80	4.90	7.20	15.80	3.60	4.30	16.30	30.40
Benzo[a]pyrene	0.40	0.40	0.70	1.90	0.60	0.70	nd	2.90
Perylene	0.90	0.80	1.20	1.40	1.60	2.40	4.80	2.10
Indeno[1,2,3-cd]pyrene	nd	nd	nd	nd	nd	nd	nd	2.70
Dibenz[a,c+a,h]anthracene	nd	nd	nd	nd	nd	nd	nd	1.70
Benzo[g,h,i]perylene	0.30	0.60	1.40	3.10	0.60	1.30	4.40	6.70
ΣPAH_18_	207.5	196.5	264.6	481.6	201.9	186.3	346.3	682.2
PHE, FLU and PYR%	83%	82%	72%	63%	82%	79%	64%	54%
LMW PAHs	80.7	75.2	97.8	169.2	92.4	85.2	106.6	197.4
HMW PAHs	126.8	121.3	166.8	312.4	109.5	101.1	239.7	484.8
LMW PAHs%	39%	38%	37%	35%	46%	46%	31%	29%
HMW PAHs%	61%	62%	63%	65%	54%	54%	69%	71%
Methylated PAHs and thioarenes								
Benzo[b]naphtho[2,1-d] thiophene	1.0	1.9	11.6	40.1	1.6	2.8	23.0	59.4
2-Methylnaphthalene	0.0	0.2	4.7	36.3	0.4	0.8	5.2	22.6
1-Methylnaphthalene	0.0	0.1	2.7	19.0	0.0	0.0	1.1	11.5
2-Methylanthracene	1.0	1.9	1.3	0.0	0.7	0.6	0.0	0.0
3-Methylphenanthrene	49.6	45.8	84.8	282.7	46.8	45.5	125.8	426.0
9-Methylphenanthrene	55.7	55.3	101.4	377.7	52.9	50.8	159.4	608.7
Dibenzothiophene (DBT)	1.0	1.6	3.2	10.9	1.8	1.8	4.9	14.0
4-Methyl-DBT	2.6	2.6	6.7	26.4	3.0	3.3	12.0	43.3
1-Methyl-DBT	1.7	1.5	5.1	22.2	1.7	1.9	8.9	34.0
3+2-Methyl-DBT	0.7	0.8	1.4	6.4	0.7	0.4	3.2	11.5
Σmethylnaphthalenes	0.0	0.3	7.4	55.3	0.4	0.8	6.3	34.1
Σmethylphenanthrenes	105.3	101.1	186.2	660.4	99.7	96.3	285.2	1034.7
ΣMethyl-DBTs	5.0	4.9	13.2	55.0	5.4	5.6	24.1	88.8
ΣPAH	320.8	308.2	487.5	1303.3	311.5	294.2	689.8	1913.2
PAH_18_% of ΣPAH	64.7%	63.8%	54.3%	37.0%	64.8%	63.3%	50.2%	35.7%
ΣMethylphenanthrenes % total PAH	32.8%	32.8%	38.2%	50.7%	32.0%	32.7%	41.3%	54.1%

*CT* control treatment, *nd* not detected

The PAHs measured in mussel tissues were divided into two groups, of which 18 PAHs (PAH_18_) belong to the compounds commonly measured in environmental samples and the other consisting of methylated PAHs and thioarenes. Of the PAH_18_, the share of the three main compounds, PHE, fluoranthene (FLU), and pyrene (PYR) was in total 83 and 80% in the control mussels (4 and 14 days, respectively), 72 and 64% in the Med group, and 63 and 54% in the Hi group. Total concentrations of PAH_18_ after 4 and 14 days were 207.5 and 201.9 ng g dw^−1^ in the control treatment, 265.0 and 346.0 ng g dw^−1^ in the Med group, and 482.0 and 682.0 ng g dw^−1^ in the Hi group, respectively. PAH concentrations did not change in the control during the duration of the exposure; therefore, the same concentration (207.5 ng g dw^−1^) was assumed to occur also at day 0 of the experiment to calculate accumulation rates for the two exposure periods (day 0 to day 4 and day 4 to day 14). In regard to the exposure concentrations, the accumulation of PAH_18_ between the start of the experiment (day 0) and day 4 was higher at both concentrations (14.3 and 68.5 ng g dw^−1^, respectively) compared to the rate between day 4 and day 14 (8.2 and 20 ng g dw^−1^). For the methylated compounds, the accumulation rates between day 0 and day 4 were 20.2 and 138 ng g dw^−1^ and from day 4 to day 14 were 9.9 and 37.4 ng g dw^−1^ in the Med and Hi, respectively. Although accumulation of PAHs continued throughout the exposure period, this comparison of daily total PAH accumulation rates calculated for day 4 and day 14 showed their ca. twofold and fourfold decline during this period in the Med and Hi exposure groups, respectively. At the same time, the share of HMW PAHs from the accumulated load is increased markedly especially in the Hi group being 71% at the end of the exposure (Table 3). Finally, examining the relationships of naphthalene (NAP) and PHE between their methylated forms in the different treatments and time points shows that the initial high ratio of the parent compound vs. the methylated form decreased rapidly during the exposure experiment (Table 3).

### Responses in *M. trossulus*

#### ADS, biotransformation, and neurotoxicity

**Fig. 1:**
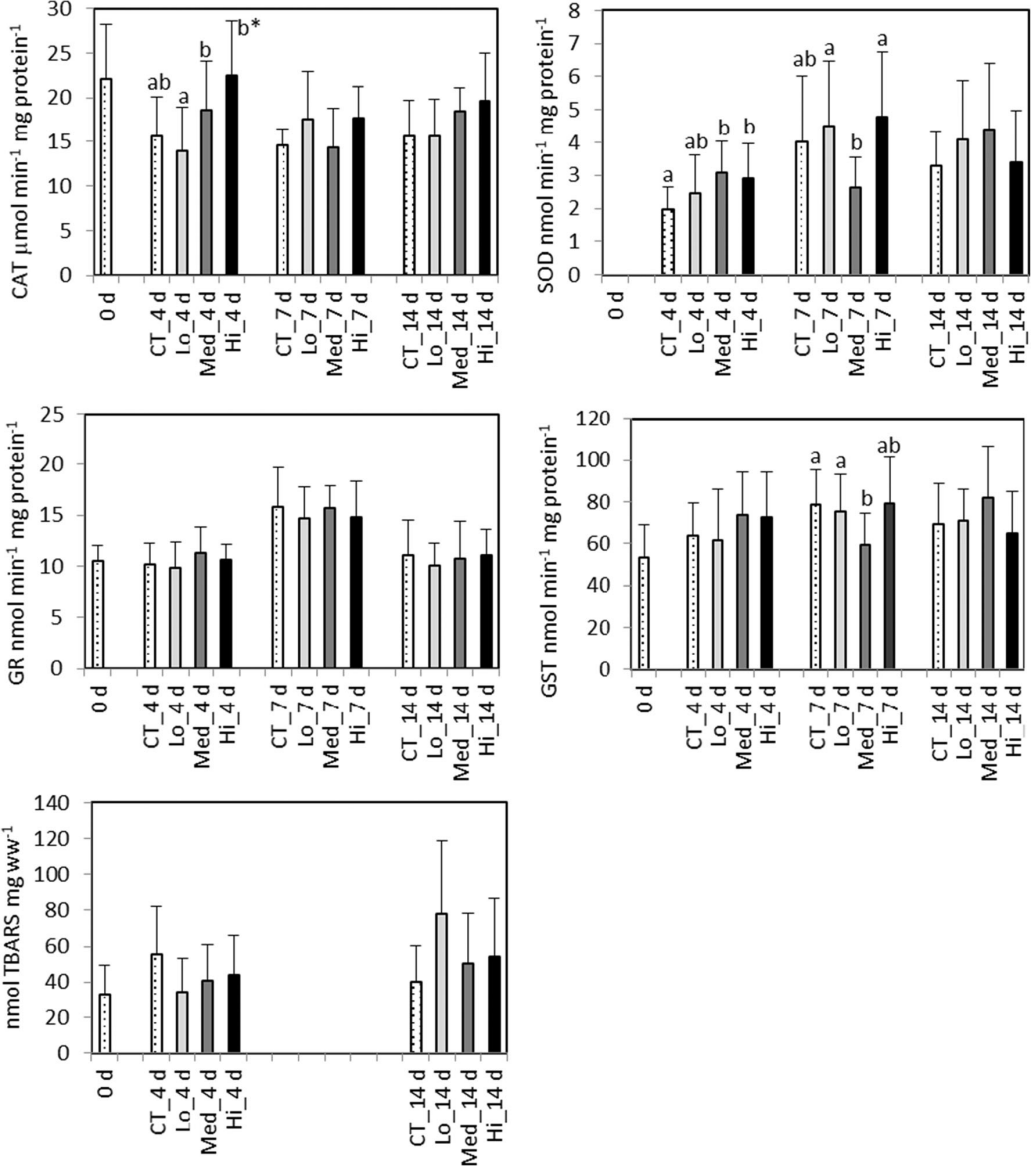
*Mytilus trossulus.* Biomarkers responses measured in the digestive gland after a 4-, 7-, and 14-day exposure to control (CT) and different concentrations of dispersed crude oil (Lo, Med, and Hi). 0 d, sampled a day before the experiment started; GST, glutathione S-transferase; CAT, catalase; GR, glutathione reductase; SOD, superoxide dismutase; LPO, lipid peroxidation. Letters denote significant differences between the groups, mean ± SD, *n* = 15, and asterisk denotes significant difference to control in CAT diagram

After 4 days of exposure, a significantly increased CAT activity in the digestive gland (*F*_3,56_ = 10.1) was observed in Hi treatment compared to the control treatment (*p* < 0.001) and in the Med treatment (*p* < 0.05) and the Hi treatment (*p* < 0.001) compared to the Lo treatment (Fig. 1). A significantly higher SOD activity (*F*_3,56_ = 4.0) was detected in the 0.120 and in Hi treatments compared to the control treatment (*p* < 0.05). In gills, CAT activity was significantly higher (*F*_3,35_ = 4.9) in the Med treatment compared to the 0.015 (*p* < 0.01) and Hi treatments (*p* < 0.02).

**Fig. 2:**
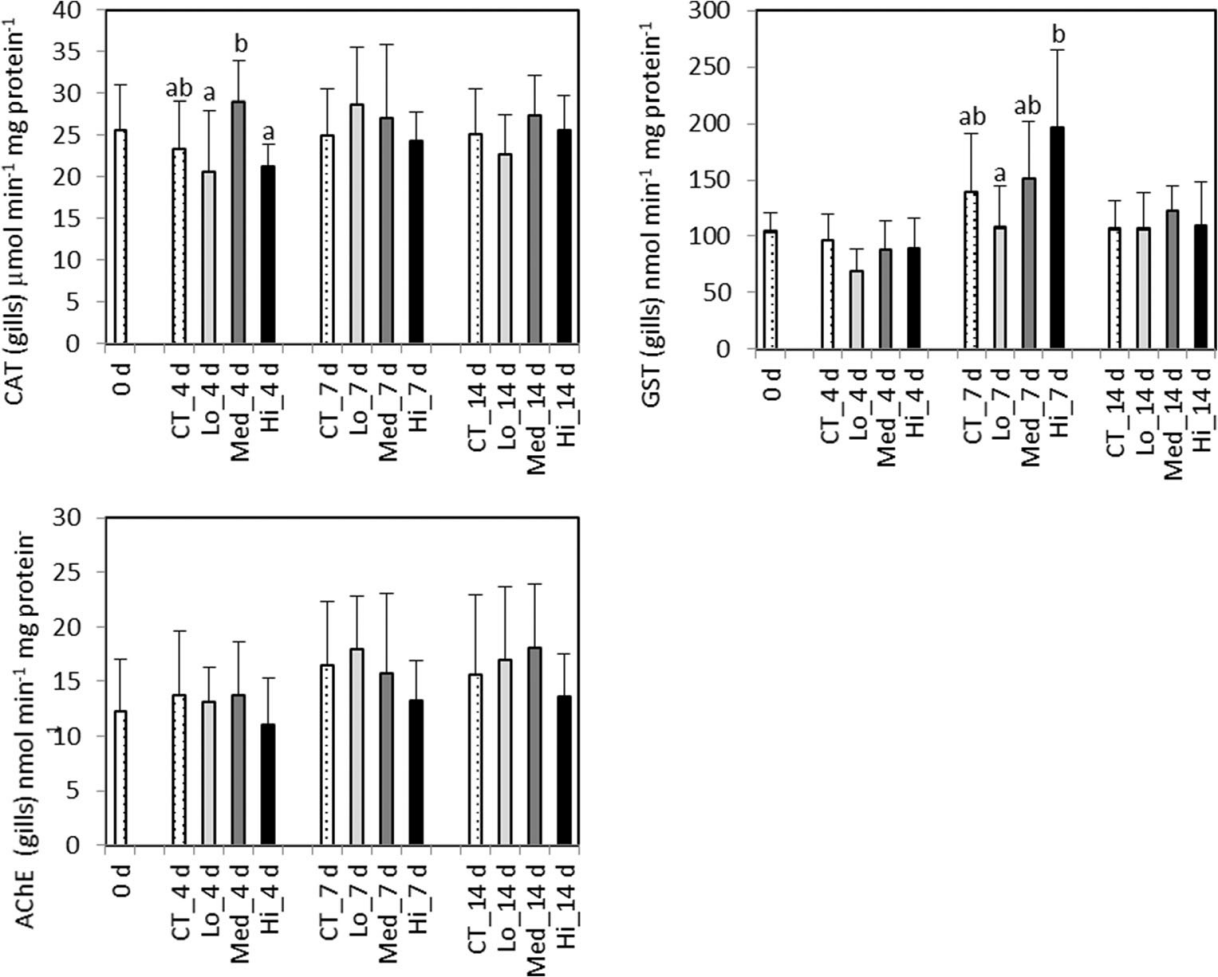
*Mytilus trossulus.* Biomarkers responses measured in the gills after a 4-, 7-, and 14-day exposure to control (CT) and different concentrations of dispersed crude oil (Lo, Med, and Hi). 0 d, sampled a day before the experiment started; GST, glutathione S-transferase; CAT, catalase; AChE, acetylcholinesterase. Letters denote significant differences between the groups, mean ± SD, *n* = 10

After 7 days of exposure, a significantly lower GST activity (*F*_3,56_ = 5.6) in the digestive gland was detected in the Med treatment compared to control (*p* < 0.001) and in the Lo (*p* = 0.03) group (Fig. 1). The activity of SOD was also significantly lower (*F*_3,56_ = 4.7) in the Med treatment compared to the 0.015 (*p* = 0.03) and Hi treatments (*p* < 0.01). In gills, the highest GST activity (*F*_3,36_ = 5.4) was detected in the Med group and it was significantly elevated compared to the Lo treatment (*p* = 0.002) (Fig. 2). After 14 days, no significant enzymatic responses to oil exposure could be detected (Fig. 2). Regarding both exposure time points, AChE activities (Fig. 2) and LPO levels (Fig. 1) did not show significant differences between treatments.

#### Morphometric body condition and weight change

**Fig. 3:**
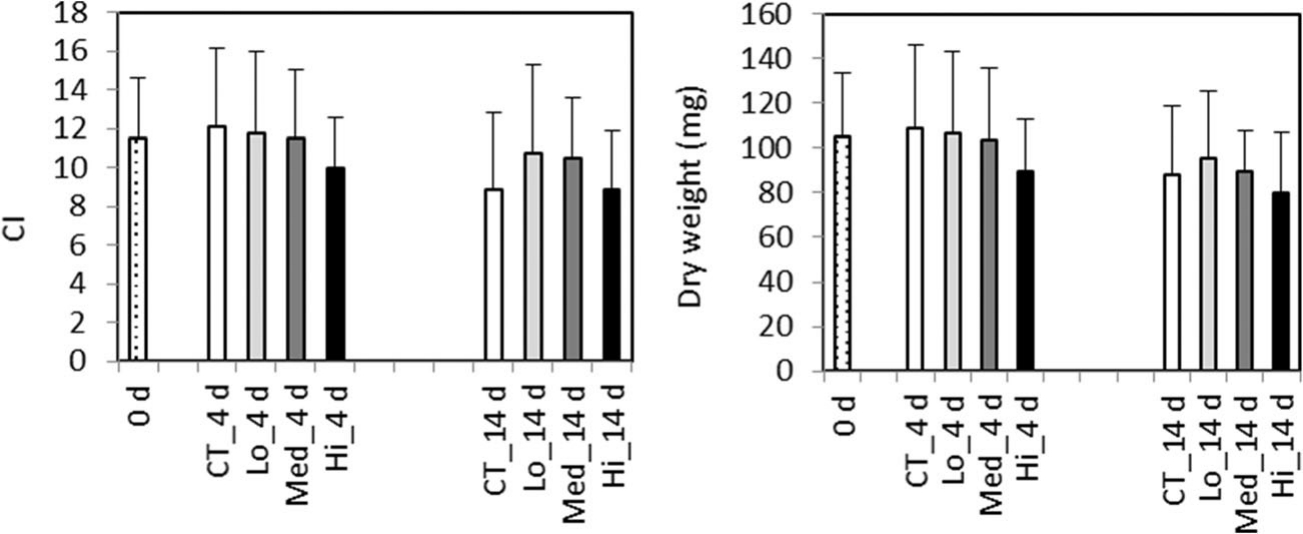
*Mytilus trossulus.* Morphometric condition index (CI) and dry weight (mg) of a “standard size” individual of 30 mm length measured at different time points of exposure to the control (CT) and different concentrations of dispersed crude oil (Lo, Med, and Hi), mean ± SD, *n* = 15

The morphometric condition index CI and the standardized soft tissue dry weight did not differ significantly between the treatments; however, a decreasing trend was observed for both parameters at day 14 compared to day 4 (Fig. 3).

#### Geno- and cytotoxicity

**Fig. 4:**
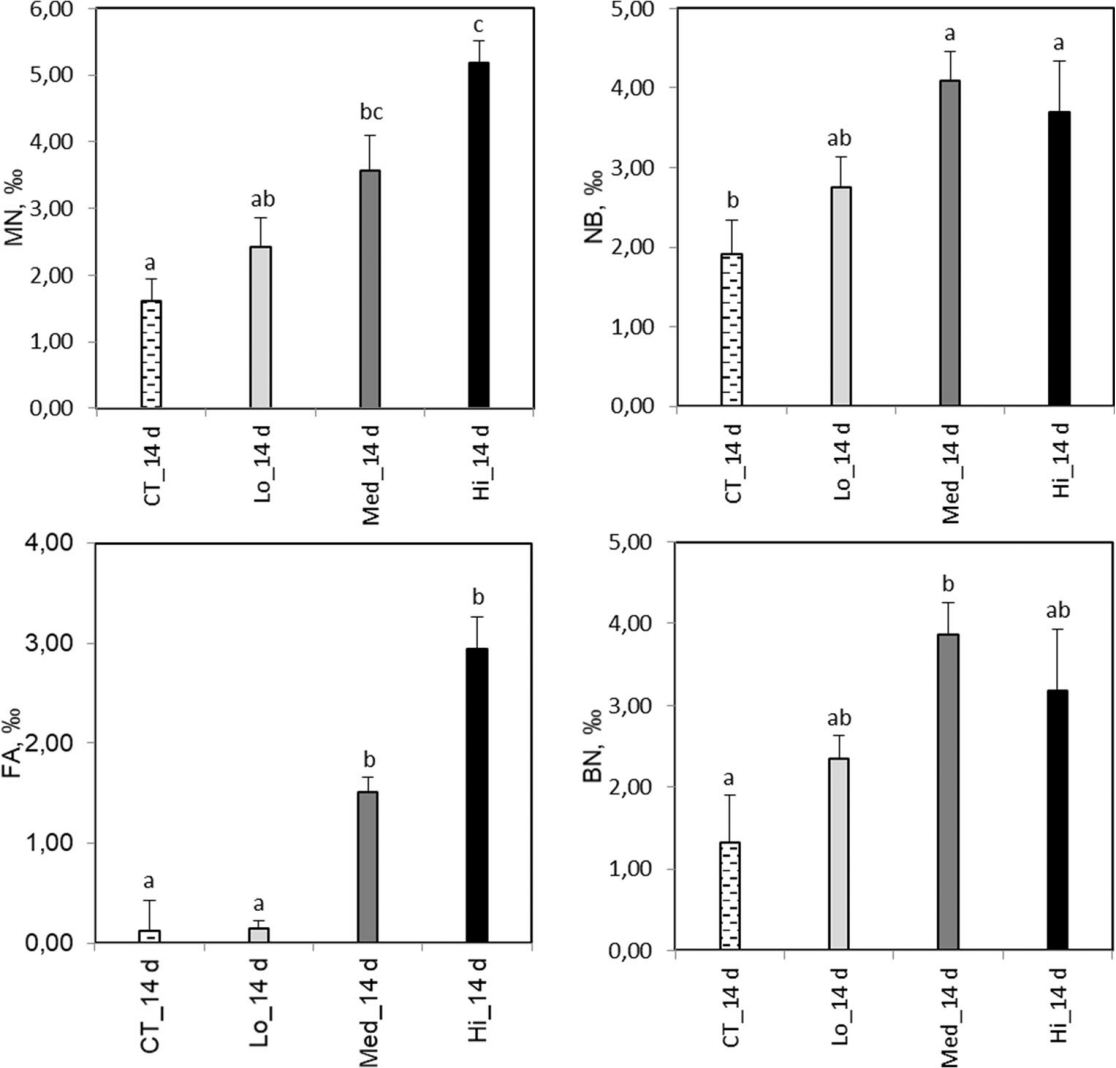
*Mytilus trossulus*. Frequencies of genotoxicity and cytotoxicity endpoints (micronuclei [MN], nuclear buds [NB], fragmented apoptotic [FA], and binucleated [BN] cells) induced in gills after exposure to control (CT) and different concentrations of dispersed crude oil (Lo, Med, Hi) for a period of 14 days. Letters denote significant differences between the groups, mean ± SEM, *n* = 10

Analyses of geno- and cytotoxicity measured as chromosomal aberrations were made only on mussels at day 14. No significant differences were found between the start and control group (data not shown). The Med and Hi treatments induced significant formation of MN (*F*_3.63_ = 12.77, *p* < 0.0001) and NB (*F*_3.63_ = 4.72, *p* = 0.005) in gill cells compared to the control group (*p* < 0.01 and *p* < 0.03, for MN and NB, respectively). Treatments with Med and Hi induced a significant formation of FA (*H* = 32.46, 3 d.f., *p* < 0.001) cells in *M. trossulus* gill cells compared to the control group (*p* = 0.007 and *p* < 0.0007, respectively). The frequency of BN (*H* = 11.17, 3 d.f., *p* = 0.011) cells showed the highest elevation in the Med treatment and the induced frequency was statistically significant (*p* = 0.01) compared to the control group. Frequencies of all the analyzed geno- and cytotoxicity endpoints did not show any significant changes in the lowest treatment group compared to control group (Fig. 4).

#### Integrated biomarker response

**Fig. 5:**
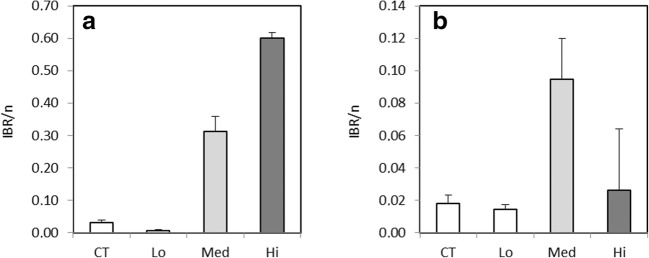
Integrated biomarker index (IBR/n) in *Mytilus trossulus* after 4 days (**a**) and 7 days (**b**) of exposure to control (CT) and to different concentrations of dispersed crude oil (Lo, Med, and Hi). Seven biomarkers (GST, CAT, GR, SOD, and LPO in the digestive gland, AChE in gills, condition index) were used for day 4 and five biomarkers (GST, CAT, GR, and SOD in the digestive gland, AChE in gills) were used for day 7 IBR index. IBR result presented as mean ± SD of three different biomarker arrangements

At day 4, the IBR index in *M. trossulus* was markedly elevated in the two highest concentrations (Fig. 5a). At day 7, the response was highest in the Med treatment, when the Hi value was already decreased due to the typical bell-shaped response curves possessed by many of the enzymatic biomarkers used here (Fig. 5b). At day 14, the calculation of the IBR with the same battery of biomarkers proved unfeasible in mussels since no differences in these biomarker responses were observed at this time point. However, continuing effect of exposure was visible in geno- and cytotoxic markers showing significant responses at day 14 in the two highest concentrations (Fig. 4).

### Responses in *G. oceanicus*

#### ADS and biotransformation

No statistical differences in the measured parameters could be detected between the replicate aquaria for each treatment and exposure time (data not shown); therefore, all the samples per treatment at each sampling point were used to calculate the final results.

**Fig. 6:**
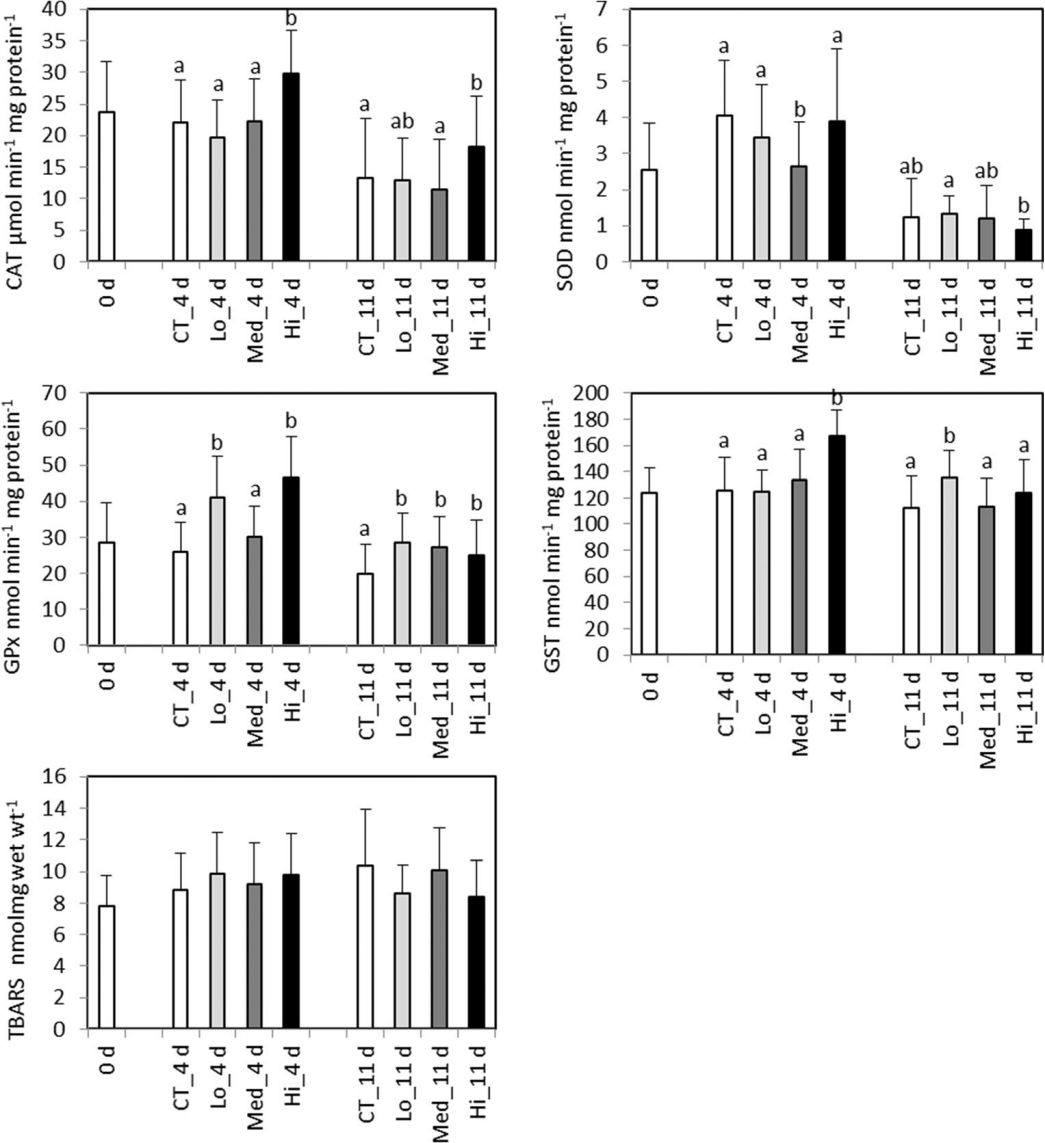
*Gammarus oceanicus.* Biomarkers responses after a 4- and 11-day exposure to control (CT) and different concentrations of dispersed crude oil (Lo, Med, and Hi). 0 d, sampled a day before the experiment started; GST, glutathione S-transferase; CAT, catalase; GPx, glutathione peroxidase; SOD, superoxide dismutase; LPO, lipid peroxidation. Letters denote significant differences between the groups, mean ± SD, *n* = 35 at 4 days and *n* = 21 at 11 days

After 4 days of exposure, the activities of GST and CAT were significantly elevated in the highest, Hi treatment compared to all the other treatments (*F*_3,73_ = 38.3, *p* < 0.0001 and *F*_3,134_ = 18.3, *p* < 0.0001, respectively; Fig. 6). The activity of GPx in the Lo and Hi treatments showed significantly higher activities compared to the control and exposure to the Med treatment (*F*_3,134_ = 34, *p* < 0.0001). SOD activity was significantly decreased at Med compared to the other treatments (*H* = 19.6, df = 3, *p* = 0.0002).

After 11 days’ exposure time, the activity of GST in the Lo group was significantly elevated compared to the other treatments (*F*_3,76_ = 4.7, *p* = 0.01; Fig. 6). A higher CAT activity was observed in the Hi treatment compared to the control and the Med group (*H* = 13.5, df = 3, *p* = 0.004). GPx activity was significantly higher in all treatments compared to the control group (*F*_3,76_ = 7.9, *p* < 0.001). A significantly lower SOD activity was detected in gammarids exposed to Hi compared to Lo (*H* = 10.1, df = 3, *p* = 0.02).

#### Integrated biomarker response

**Fig. 7:**
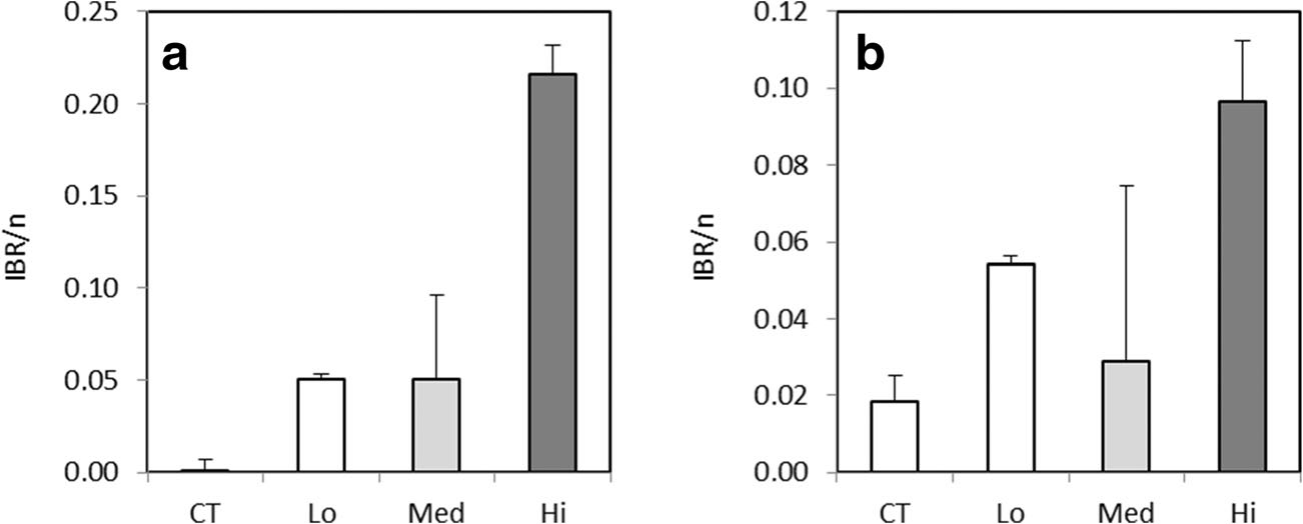
Integrated biomarker index (IBR/n) in *Gammarus oceanicus* after 4 days (**a**) and 11 days (**b**) of exposure to control (CT) and to different concentrations dispersed crude oil (Lo, Med, and Hi). Five biomarkers (GST, CAT, GPx, SOD, and LPO) were used to calculate IBR index for both time points, presented as mean ± SD of three different biomarker arrangements

On day 4 of exposure the IBR index in *G. oceanicus* was markedly higher at the 750 mg L^−1^ treatment compared to the other treatments (Fig. 7a). After 11 days of exposure, the highest response was still observed at the 750 mg L^−1^ treatment (Fig. 7b); however, it was declined due to the typical bell-shaped responses of the measured enzymes.

## Discussion

In this study, mussels and gammarid amphipods were exposed to mechanically dispersed crude oil in a concentration range considered realistic in case of the occurrence of oil spill at sea (e.g., González et al. 2006). In both species, significant biological responses were observed, supported by the accumulation patterns of oil-derived hydrocarbons measured in mussels’ tissues. The specific features and interconnections between the responses as well as their linkage to exposure concentrations, tissue accumulation of different groups of compounds, and length of exposure are discussed below.

### *Mytilus trossulus*

The fitness and survival of organisms depend on the ability to initiate a successful stress response when challenged by exposure to damaging environmental factors. In the present study, the exposed mussels accumulated most of the measured PAHs in a time- and concentration-dependent manner, except for those exposed to the lowest oil concentration of Lo, which did not show accumulation of PAHs nor significant biomarker responses compared to the control mussels. This observation is consistent with the parallel study by Ingvarsdóttir et al. (2012) on the effects of the same oil exposure arrangement using herring larvae. After 2 weeks in the highest exposure concentration of Hi, the mussels accumulated up to 682 ng g^−1^ dw of the PAH_18_ group and 1913 ng g^−1^ dw of total PAHs. In field biomonitoring studies, the highest reported total PAH concentrations measured in mussel tissues after major oil spills range between > 1000 (“Sea Empress”), 6944 (“Erika”), and 7780 ng g^−1^ dw (“Prestige”) (Neuparth et al. 2012). A high accumulation in the two highest concentrations was expected since mussels are known to eliminate PAH very slowly from their tissues (e.g., Baussant et al. 2001).

Contaminant-induced ROS overproduction directs a strong antioxidant response to prevent oxidative stress (Regoli and Giuliani 2014). A rapid (during 1–7 days) activation of the ADS as a response to oil exposure and PAH contamination has been previously described in mussels (e.g., Grintzalis et al. 2012; Livingstone 2001; Luna-Acosta et al. 2011; Orbea and Cajaraville 2006), and also in gammarids (Hatlen et al. 2009; Turja et al. 2014b).

In the present study, in mussels, increased activities of CAT and SOD were observed at day 4 during exposure to the two highest oil concentrations. In mussels, CAT activity as well as the CAT gene transcription have been shown to be rapidly increased in response to PAH exposure; however, at higher exposure concentrations especially the enzyme activity can be inhibited (Giuliani et al. 2013). The observed increase of CAT and SOD activities in mussels is consistent with the relationship between their functions (Amstad et al. 1994). Since SOD catalyzes the dismutation of the superoxide radical into H_2_O_2_, which is then detoxified by CAT and/or the GPx-GR cycle, an increase in SOD activity could be associated with an increase in H_2_O_2_ production resulting in elevated CAT activity. A positive correlation between SOD and CAT as well as positive correlation between SOD and individual tissue PAHs and total PAHs has been previously reported in mussels exposed to PAHs (Richardson et al. 2008). The apparent non-response of GR suggests that under the exposure conditions the mussels were still coping with CAT as the main H_2_O_2_ degradation enzyme. Metabolism of certain PAH compounds produces reactive intermediates as well as ROS, connecting specific biotransformation processes to the degree of oxidative challenge (Baussant et al. 2009). In the present study, an elevated GST activity was detected in the gills tissues of mussels at day 7 in the highest exposure concentration of Hi. Similarly, Baussant et al. (2009) observed a significantly increased GST activity in *Mytilus edulis* exposed to mechanically dispersed North Sea crude oil. A marked increase in the GST activity in the gills of mussels after exposure to elutriates made from “Prestige” oil residues and oil-contaminated sediment was observed by Moreira et al. (2004), while mussels collected from oil-contaminated sites showed an increased GST activity even 1 year after the spill (Moreira et al. 2004; Tim-Tim et al. 2009).

In bivalves, the potential of PHE, PYR, and FLU to induce biological responses has been extensively reported (e.g., Grintzalis et al. 2012; Moore et al. 2007; Okay et al. 2006; Richardson et al. 2008; Yakan et al. 2013). In the present study, among the accumulated individual PAH compounds, the highest tissue concentrations were detected for methylphenanthrene, triphenyl+chrysene, PHE, PYR, and FLU. Examination of temporal accumulation dynamics of PAHs showed that both the PAH_18_ group and the methylated and thioarenes group showed a markedly higher rate between day 0 and day 4 compared to the exposure period from day 4 to day 14. At day 4 in the high concentration treatment of Hi, a sixfold increase was observed in the tissue levels of 1- and 2-methylphenanthrene (sum parameter); after day 14, the tissue levels were 1.6 times higher that recorded at day 4. In bivalves, IBR index have been shown to provide indication of environmental stress caused by oil pollution (Luna-Acosta et al. 2017; Marigomez et al. 2013). In the present study, IBR index clearly showed the higher level of biological responses linked to rapid accumulation of PAHs during the first 4 days of the exposure. Prolonged exposure time resulted typical bell-shaped response curves of the measured ADS parameters and lowered IBR index values.

At 14 days of exposure, all the measured ADS responses in *M. trossulus* were at same level in the oil-exposed and control groups; however, the increased ADS response detected at day 4 in the two highest oil concentrations suggests that the oxidative challenge may already then have contributed to the formation of the nuclear abnormalities observed at day 14. Significantly elevated levels of geno- and cytotoxicity were detected in gill cells of mussels after exposure to Med and Hi concentrations. The potential of crude oil to cause geno- and cytotoxic effects for *M. trossulus* has been previously described in Baršienė and Andreikenaite (2007) showing significant MN induction in gill cells in mussels exposed to 0.05 mg L^−1^ crude oil. High levels of MN have been observed in *M. edulis* inhabiting sites heavily polluted by PAHs in the North Sea and in the Baltic Sea (Baršienė et al. 2004, 2006a). MN frequencies were significantly related to PAH concentrations measured in the mussel *Perna* from Guanabara Bay (Francioni et al. 2007). Increased levels of DNA damage have been detected in *M. edulis* and Atlantic cod (*Gadus morhua*) caged near to oil platforms in the North Sea (Hylland et al. 2008) as well as in *M. galloprovincialis* in the Adriatic Sea (Gorbi et al. 2008). A high incidence of MN after 4 and 14 days of exposure to petroleum water-soluble fraction was observed in peripheral erythrocytes of the mullet *Mugil liza* (Moreira et al. 2014). Significant long-term effects have been demonstrated in mussels showing elevated genotoxicity after 30 days post-oil spill (Parry et al. 1997) and cytogenetic damage lasting up to 6 months (Baršienė et al. 2012). Elevated levels of nuclear aberrations were also detected in the gills of *M. trossulus* caged at contaminated sites in the northern Baltic Sea (Turja et al. 2014a). Thus, the linkage of oil exposure to these geno- and cytotoxic lesions is well documented and the results frequently correlate with biomarkers related to the ADS overload (Brooks et al. 2011; Fernandez et al. 2011; Turja et al. 2014a), which leads to oxidative stress that is likely to cause macromolecular lesions, including DNA damage and subsequent chromosomal aberrations.

Examination of the tissue accumulation of PAH compounds at day 14 of the exposure showed increased share of HMW compounds compared to LMW fraction in mussel tissues in the two highest oil concentrations. This signifies a marked elevation in the accumulation of the assumedly more hazardous HMW PAHs during the exposure. Even though the uptake of LMW PAHs had practically halted at day 14 (generally observed, except for PHE), in the methylated forms it continued. It is unclear whether these changed tissue accumulation rates recorded at day 14 were due to a reduced uptake rate, activated metabolism, or depuration of the compounds. However, from a toxicological viewpoint these results are highly relevant. PHE, FLU, and PYR are by far the most commonly observed PAHs in surface waters and sediment of the Baltic Sea (Lang et al. 2015; Witt 2002) and regularly forming the majority of the background PAH levels in the tissues of mussels as well (Turja et al. 2012, 2014, 2015; Lehtonen et al. 2016). The marked decrease in the share of PHE, FLU, and PYR observed in the two highest oil treatments to the total of other PAH_18_ compounds in the tissues of mussel during the experiment clearly indicates exposure to oil, and this observation could also be useful in field studies and monitoring of oil pollution. Most of the HMW PAHs, which under exposure conditions are usually present in small quantities, show increased accumulation may be an ecologically relevant difference in case of an acute oil spill specifically in regard to the time of exposure and the onset of effects at different biological levels. Moreover, it has been recently discussed that alkylated PAHs should be more extensively studied in environmental samples since their toxic potential may easily surpass that of the parent compounds (Andersson and Achten 2015; Lam et al. 2018). For example, methylated phenanthrenes are generally recognized to be abundant among the PAHs of petrogenic origin, and they have been shown to activate the aryl hydrocarbon receptor (AhR) to induce transcriptional signaling more potently than does PHE (Sun et al. 2014). They have also been seen to be mutagenic and tumor-initiating (LaVoie et al. 1981). Thus, the investigations on the accumulation dynamics and toxicity of methylated PAHs in mussels can reveal important new information about the biological effects of oil exposure.

### *Gammarus oceanicus*

In *G. oceanicus*, the activity of CAT and GPx were significantly increased at the highest Hi treatment at day 4. Compared to ADS responses observed in mussels, the amphipod *G. oceanicus* needed to activate also the GSH pathway, most likely indicating a higher oxidative challenge caused by differences in behavioral aspects (active swimming vs. sessile mode and possibility of valve closure) and partly to the development of more severe starvation effects. In *G. oceanicus*, an increased CAT activity was previously shown in individuals exposed to 3 μg L^−1^ of B[a]P for 4 days (Turja et al. 2013). In the same study, GPx was increased in amphipods treated with 30 μg L^−1^ of B[a]P, suggesting that different H_2_O_2_ degrading pathways were activated depending on the exposure concentration. The inhibition of CAT activity under severe oxidative challenges is often partially compensated by the increased activity of the GSH pathway to remove excess H_2_O_2_ (Regoli et al. 2011). An increased GST activity was observed in the Hi treatment after 4 days of exposure compared to the other treatments. Significantly elevated GST activity has been previously reported in *G. oceanicus* exposed to B[a]P (3 μg L^−1^) for 4 days (Turja et al. 2014b), suggesting that GST plays an important role in PAH metabolism in gammarids. Regarding other crustaceans, increased GST activity has been observed in green crabs (*Carcinus maneas*) exposed to FLU (16–100 μg L^−1^) for 7 days (Rodrigues et al. 2013). In the present study, the GST response in *G. oceanicus* did not differ from the control any more after 7 days of exposure. Excessive ROS formation may inhibit GST activation, and only after they have been eliminated by other enzymes (such as CAT or GPx), a significant elevation in GST activity can take place again (Fernandes et al. 2009; Kankaanpää et al. 2007; Turja et al. 2014b). Based on this assumption, the observed lack of the GST response in *G. oceanicus* in the present study could be explained by the excess ROS indicated by the elevated CAT and GPx activities after 11 days of exposure. Highest stress indicated by the IBR value was observed at 4 and 11 days of exposure to the highest oil concentration, while exposure to lower oil concentrations resulted in less clear differences.

## Conclusions

Changing quantitative and qualitative dynamics were observed in the accumulation of PAH compounds in *M. trossulus* under a realistic oil concentration regime. In the present study, clearly elevated accumulation of all PAHs was detected during the first 4 days of exposure in the two highest exposure concentrations (Med and Hi) and was thereafter reduced significantly with marked differences in the accumulation rates for different types of PAH compounds. In the tissue concentrations of the PAH_18_, the fraction of the highly toxic HMW compounds increased markedly during the experiment as well as the tissue levels of methylated forms of PHE elevated to constitute approximately 54% of the total tissue PAH load compared to the 35% prior to the exposure. Linked with the observed higher level of biological responses, it seems clear that the rapid accumulation of PAHs during the early phase of the exposure (first 4 days) induced many of the damage preventing mechanisms (such as ADS). During prolonged exposure (from 7 up to 14 days), the ADS system could not cope with the stress, seen as typically bell-shaped response curves in the measured parameters. Biomarker responses observed in *G. oceanicus* followed a similar pattern to those recorded in *M. trossulus* up to 4 days and in the Hi treatment they maintained the elevated ADS response still at day 11. Overall, IBR index showed higher stress in exposed mussels (Med and Hi treatments) and gammarids (Hi treatment) than in the other treatments. Genotoxic and cytotoxic responses measured in mussels were present at 14 days indicating longer term effects of exposure. Moreover, toxicity of alkylated PAHs should be taken into consideration in future studies regarding oil contamination and biological effects.
